# Persons post-stroke improve step length symmetry by walking asymmetrically

**DOI:** 10.1186/s12984-020-00732-z

**Published:** 2020-08-03

**Authors:** Purnima Padmanabhan, Keerthana Sreekanth Rao, Shivam Gulhar, Kendra M. Cherry-Allen, Kristan A. Leech, Ryan T. Roemmich

**Affiliations:** 1 Center for Movement Studies, Kennedy Krieger Institute, 707 North Broadway, Baltimore, MD 21205 USA; 2 Department of Neuroscience, Johns Hopkins University School of Medicine, Baltimore, MD 21205 USA; 3 Department of Biomedical Engineering, Johns Hopkins University, Baltimore, MD 21218 USA; 4 Virginia Commonwealth University School of Medicine, Richmond, VA 23298 USA; 5 Department of Physical Medicine and Rehabilitation, Johns Hopkins University School of Medicine, Baltimore, MD 21205 USA; 6 Division of Biokinesiology and Physical Therapy, University of Southern California, Los Angeles, CA 90007 USA

**Keywords:** Gait, Stroke, Rehabilitation, Energy, Mechanics, Effort, Metabolic

## Abstract

**Background and purpose:**

Restoration of step length symmetry is a common rehabilitation goal after stroke. Persons post-stroke often retain the ability to walk with symmetric step lengths (“symmetric steps”); however, the resulting walking pattern remains effortful. Two key questions with direct implications for rehabilitation have emerged: 1) how do persons post-stroke generate symmetric steps, and 2) why do symmetric steps remain so effortful? Here, we aimed to understand how persons post-stroke generate symmetric steps and explored how the resulting gait pattern may relate to the metabolic cost of transport.

**Methods:**

We recorded kinematic, kinetic, and metabolic data as nine persons post-stroke walked on an instrumented treadmill under two conditions: preferred walking and symmetric stepping (using visual feedback).

**Results:**

Gait kinematics and kinetics remained markedly asymmetric even when persons post-stroke improved step length symmetry. Impaired paretic propulsion and aberrant movement of the center of mass were evident during both preferred walking and symmetric stepping. These deficits contributed to diminished positive work performed by the paretic limb on the center of mass in both conditions. Within each condition, decreased positive paretic work correlated with increased metabolic cost of transport and decreased walking speed across participants.

**Conclusions:**

It is critical to consider the mechanics used to restore symmetric steps when designing interventions to improve walking after stroke. Future research should consider the many dimensions of asymmetry in post-stroke gait, and additional within-participant manipulations of gait parameters are needed to improve our understanding of the elevated metabolic cost of walking after stroke.

## Introduction

Gait dysfunction is common after stroke [1]. Persons post-stroke exhibit slow walking speeds [2–4], gait asymmetry [4, 5], and an elevated metabolic cost of transport (i.e., energy expended per meter walked) [6–8]. Gait training is a key component of stroke rehabilitation, as persons post-stroke frequently list gait improvement among their most desired rehabilitation goals [9].

Many rehabilitation approaches aim to restore step length symmetry [10–16]. The rationale for restoring step length symmetry is multifaceted: 1) asymmetric stepping increases the cost of transport in healthy adults [17], 2) persons post-stroke who walk with more asymmetric step lengths also tend to exhibit poorer balance [18] and more effortful gait patterns [19], 3) step length asymmetry is a simple metric that manifests from complex kinematic and kinetic asymmetries that can be difficult to treat in isolation, and 4) step length is easy to measure and manipulate in clinical settings (e.g., “step to the lines on the floor”). Consequently, there has been increasing interest in restoring step length symmetry after stroke, especially after recent intervention studies showed that improved step length symmetry coincided with improvements in gait speed [15] and cost of transport [19].

However, it is not clear that restoration of step length symmetry alone should lead to improvements in gait speed or cost of transport. Persons post-stroke often retain the capacity to walk with improved step length symmetry, even within a single testing session [16, 20, 21]. But unlike the intervention studies mentioned above, single-session studies have shown cost of transport to be similar whether persons post-stroke walk with asymmetric or symmetric step lengths [16, 21]. These findings suggest that improvements in gait speed and cost of transport likely arise from changes in kinematic or kinetic parameters that more directly influence gait speed or energetics and also affect step length symmetry. From this perspective, interventions that aim to restore step length symmetry but do not affect these critical underlying factors may not result in meaningful gait improvement. The ability to lessen cost of transport with an intervention aiming to restore step length symmetry likely depends on 1) the underlying causes of the asymmetry (which vary among patients [21, 22]), and 2) the mechanics used to generate the symmetric step lengths.

Here, we aimed to understand how persons post-stroke changed their walking patterns to restore step length symmetry and how these gait mechanics related to the cost of transport. We asked: do persons post-stroke restore step length symmetry by restoring symmetric gait mechanics or by relying on asymmetric compensatory mechanics? We hypothesized that persons post-stroke would restore step length symmetry using asymmetric walking patterns. We then aimed to explain why these asymmetric gait patterns cost so much energy despite improved step length symmetry.

## Materials and methods

### General methods

Ten persons post-stroke were recruited for the study. Data accrued from nine persons were retained for analysis (6 M/3F, age (mean ± SEM): 54 ± 4 years, lower extremity Fugl-Meyer [23]: 26 ± 1, body mass: 93 ± 6 kg, all > 6 months post-stroke). Inclusion criteria for recruitment included a step length difference of at least 2 cm during over-ground walking. One participant was excluded from analysis because they unexpectedly reduced the asymmetry below 2 cm during treadmill walking. All other participants showed a > 2 cm step length difference during both over-ground and treadmill walking and reduced their step length asymmetry from the preferred walking trial to the symmetric stepping trial. Participants reported no additional neurological, musculoskeletal, or cardiovascular conditions. We determined preferred walking speed as the average speed of three over-ground 10-meter walk tests (0.81 ± 0.09 m/s, range: 0.40–1.25 m/s). Seven participants held onto the treadmill handrails, two wore ankle-foot orthoses, and one received functional electrical stimulation of the tibialis anterior. We asked participants who held onto the handrails to hold onto them as little as possible and avoid gripping the handrail if at all possible. All participants wore a safety harness that did not provide body weight support, provided written informed consent in accordance with the Johns Hopkins Medicine Institutional Review board prior to participation, and received monetary compensation.

**Fig. 1:**
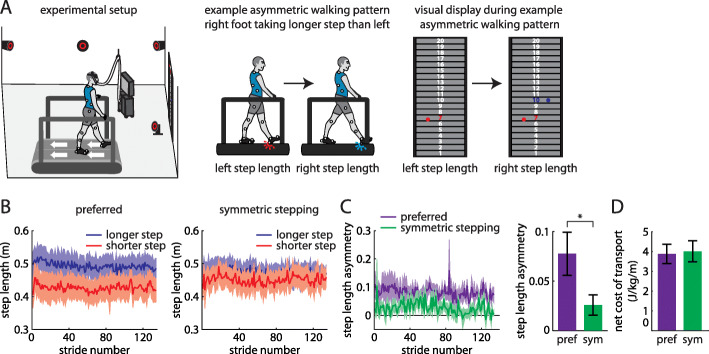
**a** Experimental setup (left). Example participant walking with asymmetric step lengths (center) and resulting visual display showing step length feedback bilaterally (right). **b** Step lengths (mean ± SE curves) for the limbs that took longer (blue) and shorter (red) steps at baseline during preferred walking (left) and symmetric stepping (right). The data shown have been truncated to number of strides for the participant that took the fewest strides for the same duration of the trial. **c** Step length asymmetry decreases significantly during symmetric stepping (green) as compared to preferred walking (purple). **d** The net metabolic cost of transport is similar between preferred walking and symmetric stepping

We recorded kinematic (100 Hz) and kinetic (1000 Hz) data using a three-dimensional motion capture system (Vicon, Oxford, UK) and instrumented split-belt treadmill (Motek, Amsterdam, NL; Fig. 1a, left). We placed retroreflective markers over the seventh cervical vertebrae, tenth thoracic vertebrae, jugular notch, xiphoid process, and bilaterally over the second and fifth metatarsal heads, calcaneus, medial and lateral malleoli, shank, medial and lateral femoral epicondyles, thigh, greater trochanter, iliac crest, and anterior and posterior superior iliac spines. We filtered marker trajectories and ground reaction forces (GRFs) with fourth order low-pass Butterworth filters (6 Hz and 15 Hz cut-off frequencies, respectively). GRFs were set to zero for vertical GRF magnitudes < 32 N. Participants wore comfortable shoes and form-fitting clothing.

We collected metabolic data using a TrueOne 2400 system (Parvomedics, Sandy, UT) that warmed up for > 30 min before data collection and was calibrated to manufacturer specifications. We sampled oxygen consumption and carbon dioxide production breath-by-breath. We collected 2 minutes of baseline metabolic data during quiet standing. We used a traditional equation [24] to calculate total metabolic power during walking trials and subtracted baseline metabolic power to calculate net metabolic power. We calculated net metabolic cost of transport (herein referred to as cost of transport) by normalizing net metabolic power to treadmill speed.

### Visual display

Our feedback display showed 20 vertically-arranged virtual targets on a 4 m × 2.5 m screen in front of the treadmill [16, 25]. The targets provided a reference frame for participant step lengths. Each target was 2.5 cm wide, and the step lengths associated with specific targets were adjusted for each participant’s preferred step lengths to ensure that the virtual step length markers never disappeared from the screen (i.e., spanned above or below the target board). For example, the step length associated with target “1” changed among participants depending on their step lengths, but all participants had to lengthen their step lengths by 2.5 cm to move a marker from target “1” to target “2”. Red and blue circles appeared on the left and right halves of the display, respectively, at heel-strikes (detected in real-time using force plates) to represent left and right step lengths (Fig. 1a, middle and right). The white number centered inside the target changed color (red at left heel-strike, blue at right heel-strike) when it had been reached with each foot.

### Protocol

Participants performed three treadmill walking trials, each 4 minutes in duration, at preferred speeds. Participants first walked without feedback (baseline). This enabled us to measure baseline asymmetry and identify which leg took a longer step. We then displayed step length feedback, and participants walked with their preferred gait pattern or symmetric step lengths (order randomized). During preferred walking, participants received visual feedback about their step lengths but were not instructed to use the feedback and walked normally. During symmetric stepping, we asked participants to hit the same target with each pair of steps. We did not enforce constraints on individual step lengths or provide instructions about which leg should step longer or shorter to improve step length symmetry.

### Spatiotemporal and kinematic measurements

We measured step length as the distance between the lateral malleoli markers along the anterior-posterior axis at heel-strike and step length asymmetry as the difference in consecutive step lengths between the leg that took a longer step at baseline and the leg that took a shorter step, normalized to their sum:

$$ step\ length\ asymmetry=\frac{\left({step\ length}_{longer}-{step\ length}_{shorter}\right)}{\left({step\ length}_{longer}+{step\ length}_{shorter}\right)} $$

We also developed a measure of kinematic asymmetry – interlimb asymmetry (IA) - that was agnostic to each participant’s idiosyncratic gait deficits. This was important in enabling us to understand whether the participants improved step length symmetry with asymmetric kinematics without assigning the asymmetries to specific joints. Furthermore, we could determine whether the kinematic patterns used to improve symmetric step lengths were similar to participants’ preferred walking patterns.

IA quantifies asymmetry in individual limb segment contributions to step length. For example, consider a right step length of 0.5 m. If the distance between the left lateral malleolus and lateral femoral epicondyle markers is 0.05 m at right heel-strike, the trailing (left) shank segment contribution to step length is 0.05 m/0.5 m, or 0.10. These segment contributions were calculated along the anterior-posterior axis for the following segments and sum to 1:

- trailing shank (trailing lateral malleolus to lateral femoral epicondyle, *a*)
- trailing thigh (trailing lateral femoral epicondyle to greater trochanter, *b*)
- trailing pelvis (trailing greater trochanter to iliac crest, *c*)
- trailing contribution from pelvic rotation (trailing iliac crest to center of pelvis, *d*)
- leading contribution from pelvic rotation (center of pelvis to leading iliac crest, *e*)
- leading pelvis (leading iliac crest to greater trochanter, *f*)
- leading thigh (leading greater trochanter to lateral femoral epicondyle, *g*)
- leading shank (leading lateral femoral epicondyle to lateral malleolus, *h*)

We calculated IA by summing the segment asymmetries between the left (*l*) and right (*r*) legs at consecutive heel-strikes (i.e., absolute values of the differences between each segment contribution bilaterally):

$$ interlimb\ asymmetry\ (IA)=\left|{a}_{l, lhs}-{a}_{r, rhs}\right|+\left|{b}_{l, lhs}-{b}_{r, rhs}\right|+\left|{c}_{l, lhs}-{c}_{r, rhs}\right|+\left|{d}_{l, lhs}-{d}_{r, rhs}\right|+\left|{e}_{l, lhs}-{e}_{r, rhs}\right|+\left|{f}_{l, lhs}-{f}_{r, rhs}\right|+\left|{g}_{l, lhs}-{g}_{r, rhs}\right|+\left|{h}_{l, lhs}-{h}_{r, rhs}\right| $$

IA is bounded between 0 (completely symmetric segment contributions) and 2 (completely asymmetric segment contributions). This metric can remain constant across different step lengths as long as the segment contributions scale proportionally.

### Kinetic measurements

We calculated the instantaneous center of mass (COM) mechanical power using the individual limbs method [26] as has been done previously in persons post-stroke [27, 28]. This method assumes a mechanical model of gait that allows for calculation of instantaneous COM power generated by each leg as the dot product of the GRF vector of each leg and the COM velocity vector [26]. We performed these calculations for the last five strides of each condition that exhibited clean force plate strikes (i.e., each foot landed on a different force plate). Each stride began with a paretic limb heel-strike. We partitioned each stride into four periods (two step-to-step transition periods and two non-transition periods) based on time points when the COM velocity vector was redirected within the sagittal plane [29]. The onset of the first period was considered the cessation of the fourth period of the previous stride. We calculated the positive and negative work done on the COM by each limb during the four periods by integrating the positive and negative portions of the power curve within each period. All kinetic measures were normalized to body mass.

### Data analysis

We averaged step length asymmetry, individual step lengths, IA, and segment asymmetries across the four-minute trial for each participant and condition. We calculated cost of transport over the final minute of each trial to ensure steady state measurement. In our GRF and COM velocity analyses, we set the anterior-posterior (AP) axis to be positive in the forward direction, mediolateral (ML) axis to be positive in the direction from the paretic limb toward the nonparetic limb, and vertical axis to be positive upward. We calculated GRF peaks as the most positive values produced along each axis by each leg (except the nonparetic ML GRF peak, which was calculated as the most negative value) stride-by-stride over the final five clean strides. We identified peak COM velocities at two time points. We calculated peak AP and vertical COM velocities as the most positive velocities observed when the corresponding GRF magnitude was also positive. We calculated peak ML COM velocities as the most positive COM velocity when the paretic ML GRF was positive (paretic) and the most negative velocity when the nonparetic ML GRF was negative (nonparetic). We calculated positive and negative work done by each limb during each of the four gait cycle periods stride-by-stride over the final five clean strides and then averaged across strides for each participant and condition. We also calculated total positive and negative work done by each limb across the gait cycle stride-by-stride over the final five clean strides and then averaged across strides for each participant and condition.

### Statistical analysis

We performed paired t-tests to compare step length asymmetry, cost of transport, and IA between conditions (preferred walking and symmetric stepping) and step length symmetry with and without feedback. We performed a 2 × 2 step (shorter, longer) x condition (preferred walking, symmetric stepping) repeated measures ANOVAs to compare changes in step lengths. We performed a 7 × 2 limb segment (*a* through *h* as described above, with *d* and *e* summed to indicate pelvic rotation) x condition repeated measures ANOVA to compare segment asymmetry among segments and between conditions. We performed 2 × 2 leg (paretic, nonparetic) x condition repeated measures ANOVAs to compare GRF peaks, COM velocity peaks, and positive and negative work done across legs and conditions. We performed Pearson’s correlations to assess the following relationships: step length symmetry vs. cost of transport, IA during preferred walking vs. IA during symmetric stepping, IA vs. cost of transport, cost of transport vs. positive paretic and nonparetic work, preferred walking speed vs. positive paretic and nonparetic work, and IA vs. positive paretic and nonparetic work. We set α ≤ 0.05, performed Mauchly’s tests of sphericity (Greenhouse-Geisser corrections were applied when sphericity was violated), and applied post hoc corrections for multiple comparisons where appropriate (Bonferroni for analyses with three comparisons, Dunn-Sidak otherwise).

## Results

### Persons post-stroke can walk with more symmetric step lengths, but this does not change the cost of transport

All participants walked with asymmetric step lengths during preferred walking (Fig. 1b, left) and successfully adjusted their step lengths during the symmetric stepping condition (Fig. 1b, right) to reduce step length asymmetry (t (8)=3.99, *p* < 0.01; Fig. 1c). When comparing step lengths across leg and conditions, we expectedly observed a significant main effect of leg (F (1, 8)=18.264, *p* = 0.003) and a significant interaction (F (1, 8)=33.72, *p* < 0.001). Post-hoc analyses revealed that there was a significant increase in step length in the shorter limb (*p* = 0.02) and a significant decrease in step length in the longer limb (*p* = 0.04) from the preferred to symmetric stepping conditions. We replicated prior findings [16, 21] showing that improving step length symmetry with visual feedback had no significant effect on cost of transport (t (8)=0.92, *p* = 0.38; Fig. 1d). Furthermore, when comparing the preferred walking trials with feedback off versus feedback on, the presence of visual feedback did not affect step length asymmetry (t (8)=0.07, *p* = 0.95) or metabolic cost (t (8)=0.49, *p* = 0.64). Importantly, we also did not observe any between-participant correlation between step length symmetry and increased cost of transport in either condition (preferred walking, *r* = − 0.22, *n* = 9, *p* = 0.53; symmetric walking, *r* = − 0.32, *n* = 9, *p* = 0.36).

### Persons post-stroke exhibit marked IA even when walking with symmetric step lengths

**Fig. 2:**
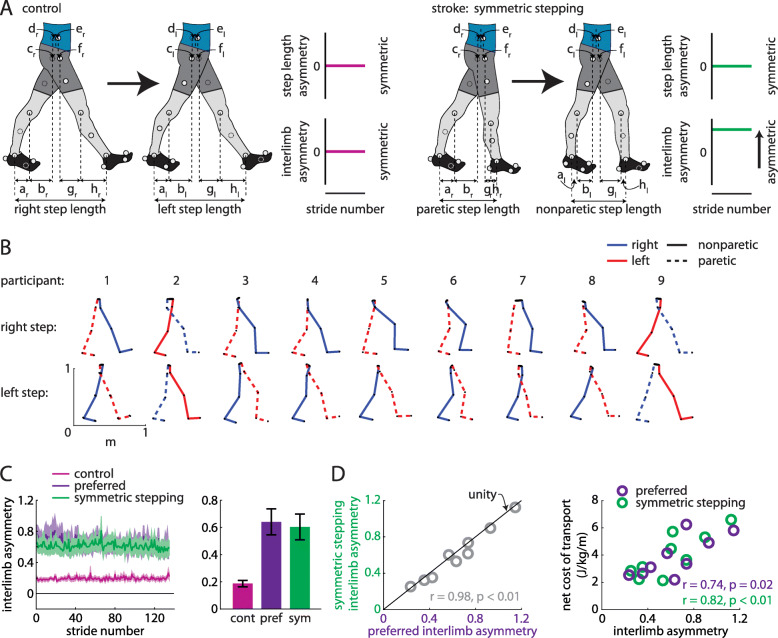
**a** Hypotheses regarding walking patterns used by control (healthy young adults) and persons post-stroke to achieve step length symmetry. We hypothesized that healthy adults achieve step length symmetry using symmetric kinematics (as represented by small interlimb asymmetry (IA); left) and persons post-stroke achieve step length symmetry using asymmetric kinematics (large IA; right). **b** Limb orientations (blue = right, red = left, solid = nonparetic, dashed = paretic) during representative steps for each participant. **c** Persons post-stroke show marked IA during preferred walking and symmetric stepping (mean ± SEM). Control data shown for reference. **d** IA during symmetric stepping correlates strongly with IA during preferred walking in persons post-stroke (left). Net metabolic cost of transport correlates strongly with IA during preferred walking and symmetric stepping (right)

A conceptual illustration of how we expected IA may differ between healthy symmetric walking and symmetric stepping after stroke is shown in Fig. 2a. We hypothesized that healthy walking consists of symmetric step lengths and similar contributions of each segment to step lengths bilaterally, resulting in small IA (Fig. 2a, left). On the contrary, we expected that symmetric stepping after stroke consists of symmetric step lengths but asymmetric segment contributions, resulting in high IA (Fig. 2a, right).

We show the limb segment orientations during representative steps of symmetric stepping for each participant (Fig. 2b). We did not observe a significant reduction in IA during symmetric stepping as compared to preferred walking (t (8)=2.12, *p* = 0.066; Fig. 2c). While it is possible that there is a trend toward a mild decrease in IA with symmetric step lengths, IA during symmetric stepping remained markedly increased when compared to healthy symmetric gait (for reference, data from eight healthy adults (age: 26 ± 5 years) walking at 1.25 m/s are shown in Fig. 2c). IA during preferred walking correlated strongly with IA during symmetric stepping (*r* = 0.98, *n* = 9, *p* < 0.01; Fig. 2d, left) and, qualitatively, the data fell near the unity line (Fig. 2d, left), suggesting that persons post-stroke showed similar IA during preferred walking and symmetric stepping. IA was significantly associated with cost of transport during preferred walking (*r* = 0.74, *n* = 9, *p* = 0.02) and symmetric stepping (*r* = 0.82, *n* = 9, *p* < 0.01; Fig. 2d, right), suggesting that kinematic asymmetries are correlated with cost of transport regardless of step length asymmetry.

**Fig. 3:**
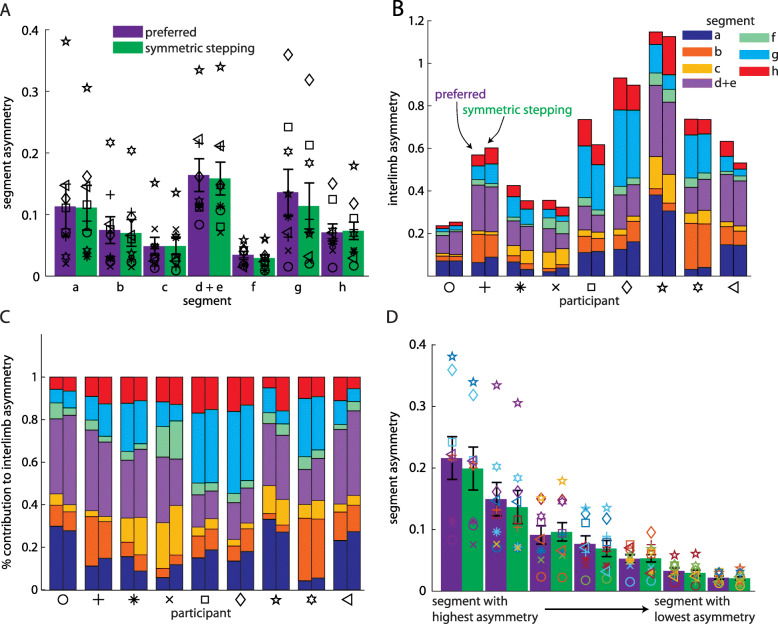
**a** Asymmetries in individual segment contributions to IA during preferred walking and symmetric stepping (organized by segment; mean ± SEM). Symbols represent individual participants. **b** Individual segment contributions to IA during preferred walking and symmetric stepping (organized by participant). For each pair of bars, the preferred walking data are represented by the left bar and the symmetric stepping data by the right. **c** Individual segment contributions to IA during preferred walking and symmetric stepping shown as a percentage of IA. **d** Asymmetries in individual segment contributions to IA during preferred walking and symmetric stepping (segments organized from highest asymmetry to lowest asymmetry). Symbols follow same scheme as in **a**-**c** and symbol colors follow the same scheme as in **b** and **c**

We next considered that IA could remain similar across conditions while individual segment asymmetries could be reorganized. We did not find this to be the case. We compared the individual segment asymmetries (e.g., |*a*_*l*, *lhs*_ − *a*_*r*, *rhs*_|) across segments and between conditions. ANOVA revealed a significant main effect of segment (F(2.312,18.497) = 4.87, *p* = 0.017). Post hoc analyses revealed that segment asymmetry was significantly larger in pelvic rotation (*d + e*) than both leading (*f*; *p* = 0.022) and trailing pelvis translation (*c*; *p* = 0.023). We did not observe a significant main effect of condition (F (1, 8)=4.49, *p* = 0.067; Fig. 3a) or segment x condition interaction (F(2.03,16.25) = 0.6, *p* = 0.49). Figure 3b and c show how the segment asymmetries contribute to IA for each participant during each condition. When we compared segment asymmetries after ordering them by which contributed most-to-least strongly to IA (during preferred walking) between conditions, we also did not observe a significant main effect of condition (F (1, 8)=4.49, *p* = 0.067; Fig. 3d) or segment x condition interaction (F(2.5,19.98) = 2.153, *p* = 0.134). As expected, we observed a significant main effect of segment (F(1.5,12.06) = 28.38, *p* < 0.001).

### Asymmetries in AP GRFs, ML GRFs, and vertical COM velocities observed during preferred walking persist during symmetric stepping

We then aimed to identify the features of these asymmetric walking patterns that may influence the elevated cost of transport regardless of step length asymmetry. We investigated whether these features were similar in both preferred walking and symmetric stepping, or whether the costs of transport were similarly high in these conditions but resulted from different underlying mechanics. Asymmetric kinematics at heel-strike should result in asymmetric mechanical work done on the COM by each leg, and previous studies demonstrated that mechanical work done on the COM is related to cost of transport in healthy adults [26, 30]. Furthermore, prior studies identified periods of the gait cycle where excessive positive work is often observed post-stroke, contributing to an elevated mechanical energetic cost [7, 8, 31].

**Fig. 4:**
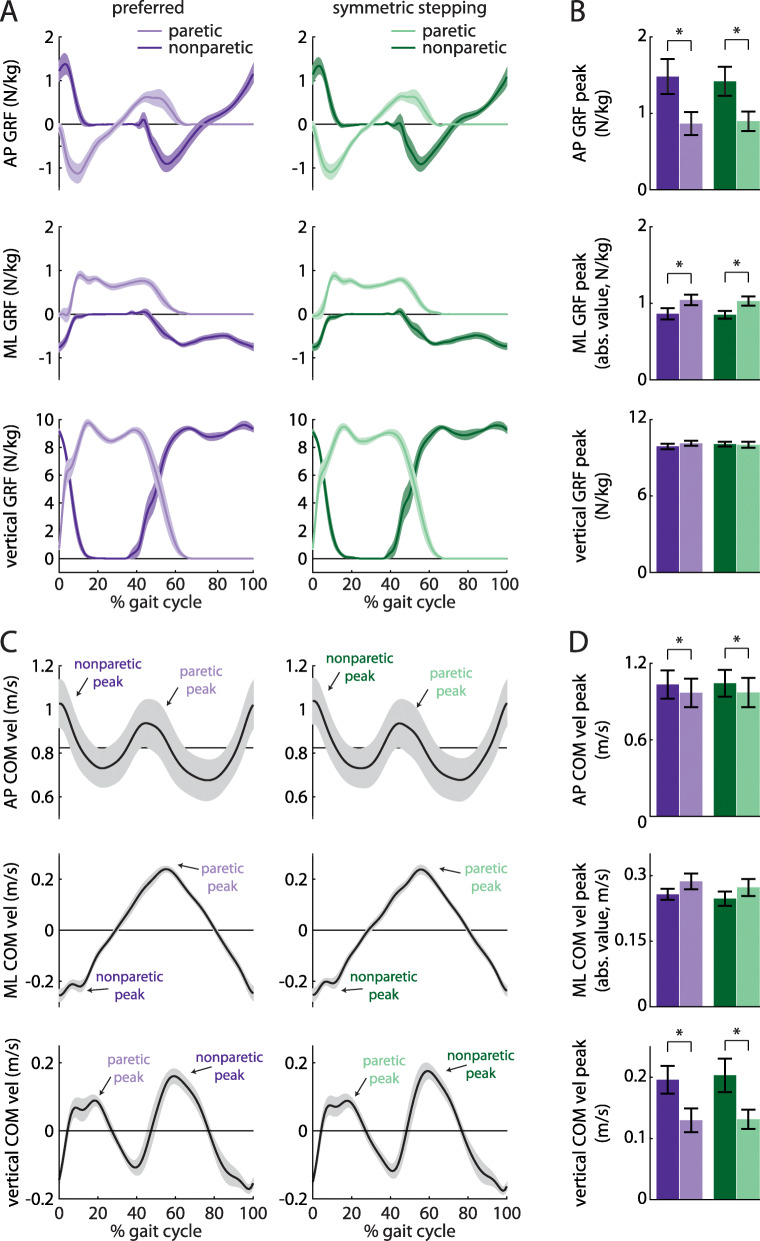
**a** Anterior-posterior (AP; top), mediolateral (ML; middle), and vertical (bottom) ground reaction force (GRF) profiles for the paretic (light colors) and nonparetic (dark colors) limbs during preferred walking (purple) and symmetric stepping (green). The gait cycle is aligned to paretic heel-strike. Persons post-stroke show decreased peak AP force production and increased peak ML force production in the paretic limb during both conditions. **b** Summary data for GRF peaks showing mean ± SEM. **c** AP (top), ML (middle), and vertical (bottom) center of mass (COM) velocity profiles during preferred walking and symmetric stepping. Peaks are labeled ‘paretic’ or ‘nonparetic’ based on the leg that most strongly contributed to the velocity. Persons post-stroke show increased AP and vertical COM velocity during late paretic stance as compared to late nonparetic stance during both conditions. **d** Summary data for COM velocity peaks showing mean ± SEM. *p < 0.05 between limbs

We investigated GRF and COM velocity profiles between legs and conditions, as these contribute to the work done over the gait cycle. ANOVA revealed a main effect of leg on the AP GRF peak (Fig. 4a and b, top; F (1, 8)=10.29, *p* = 0.01), ML GRF peak (Fig. 4a and b, middle; F (1, 8)=7.55, *p* = 0.03), AP COM velocity peak (Fig. 4c and d, top; F (1, 8)=8.53, *p* = 0.02), and vertical COM velocity peak (Figs. 4c and d, bottom; F (1, 8)=6.63, *p* = 0.03). Post hoc analyses revealed that the AP GRF peak was significantly larger in the nonparetic leg than the paretic leg (*p* = 0.01), the ML GRF peak was significantly larger in the paretic leg than the nonparetic leg (*p* = 0.03), the AP COM velocity peak was significantly larger during nonparetic late stance as compared to paretic late stance (*p* = 0.02), and the vertical COM velocity peak was significantly larger during paretic late stance as compared to nonparetic late stance (*p* = 0.03). There were no significant effects of leg on the vertical GRF peak (F (1, 8)=0.43, *p* = 0.53) or ML COM velocity peak (F (1, 8)=2.80, *p* = 0.13). We did not observe significant effects of condition on GRF or COM velocity variables (all *p* > 0.17) or leg x condition interactions (all *p* > 0.31).

### The nonparetic leg does more positive work than the paretic leg during preferred walking and symmetric stepping

**Fig. 5:**
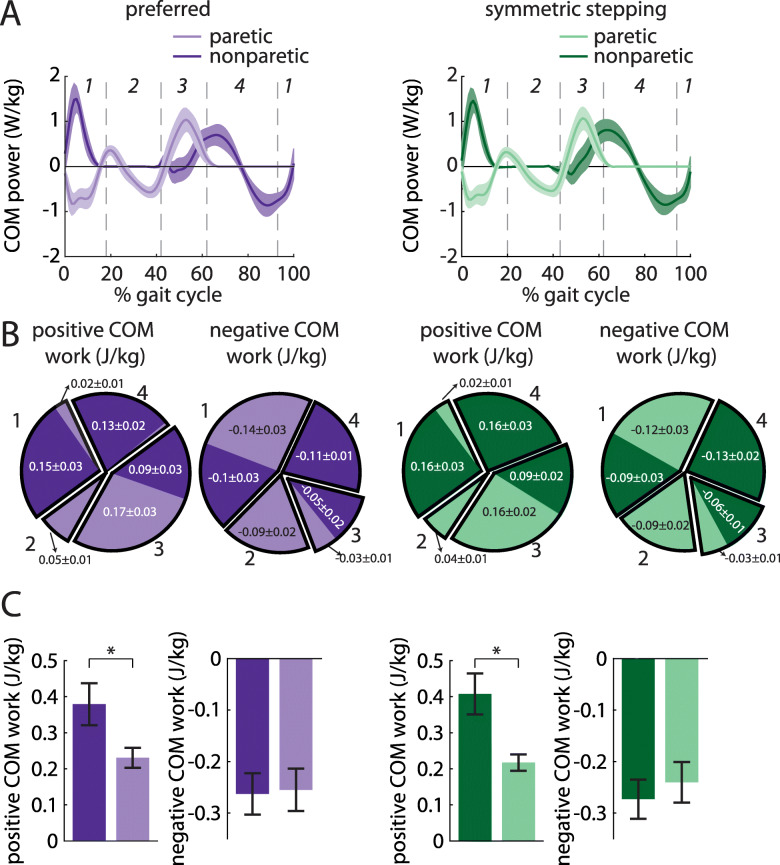
**a** COM power (mean ± SEM) generated by the paretic (light colors) and nonparetic (dark colors) limbs during preferred walking (left) and symmetric stepping (right). Gait cycles are aligned to paretic heel-strike and partitioned into four periods defined by changes in direction of the COM velocity vector within the sagittal plane. **b** Positive and negative COM work (mean ± SEM) performed by each limb in the four periods during preferred walking (left) and symmetric stepping (right). Pie charts display fractions of overall positive or negative work during each period in each condition. Numerical labels on individual limb work contributing to less than 1% of total mechanical work in each condition and results of statistical analyses are omitted for clarity. **c** Total positive and negative COM work (mean ± SEM. *p < 0.05 between limbs) performed by each limb across all four periods during preferred walking (left) and symmetric stepping (right). Persons post-stroke perform more positive work with the nonparetic limb than the paretic limb during both conditions

We next investigated the work done on the COM by each leg across conditions. We first calculated COM power for each leg during preferred walking and symmetric stepping (Fig. 5a). We calculated COM work by integrating COM power over each of the four time periods described in the methods (Fig. 5b and c). ANOVA revealed a significant main effect of leg on positive work done 1) by the paretic leg during the first period (step-to-step transition, nonparetic leg trailing) vs. the nonparetic leg during the third period (step-to-step transition, paretic leg trailing; F (1, 8)=10.96, *p* = 0.01), and 2) by the paretic leg during the second period (paretic single support) vs. the nonparetic leg during the fourth period (nonparetic single support; F (1, 8)=11.85, *p* < 0.01). Post hoc analyses revealed that the nonparetic leg did significantly more positive work during the third period than the paretic leg did during the first period (*p* = 0.01). The nonparetic leg also did significantly more positive work during the fourth period than the paretic leg did during the second period (*p* < 0.01).

ANOVA also revealed a significant main effect of leg on negative work done 1) by the paretic leg during the first period (step-to-step transition, nonparetic leg trailing) vs. the nonparetic leg during the third period (step-to-step transition, paretic leg trailing; F (1, 8)=6.35, *p* = 0.04), and 2) by the paretic leg during the third period vs. the nonparetic leg during the first period (F (1, 8)=7.06, *p* = 0.03). Post hoc analyses revealed that the paretic leg did significantly more negative work during the first period than the nonparetic leg did during the third period (*p* = 0.04). However, the nonparetic leg did significantly more negative work during the first period than the paretic leg did during the third period (*p* = 0.03). Note on Fig. 5a that the first transition period begins prior to paretic heel-strike at approximately 95% of the prior gait cycle.

We did not observe a significant main effect of condition on work done over any of the time periods (all *p* > 0.13). We did observe a significant leg x condition interaction for the positive work done during the fourth period (nonparetic single support; F (1, 8)=7.43, *p* = 0.03); however, post hoc analyses did not reach statistical significance.

A separate ANOVA revealed a significant main effect of leg on positive (but not negative; F (1, 8)=0.03, *p* = 0.87) work done across all time periods (F (1, 8)=7.25, *p* = 0.03). We did not observe a significant main effect of condition on positive or negative work done across all time periods (both *p* > 0.57) nor did we observe a significant leg x condition interaction on positive or negative work done across all periods (both *p* > 0.10).

### Less positive work done by the paretic leg is correlated with higher cost of transport and slower walking

**Fig. 6:**
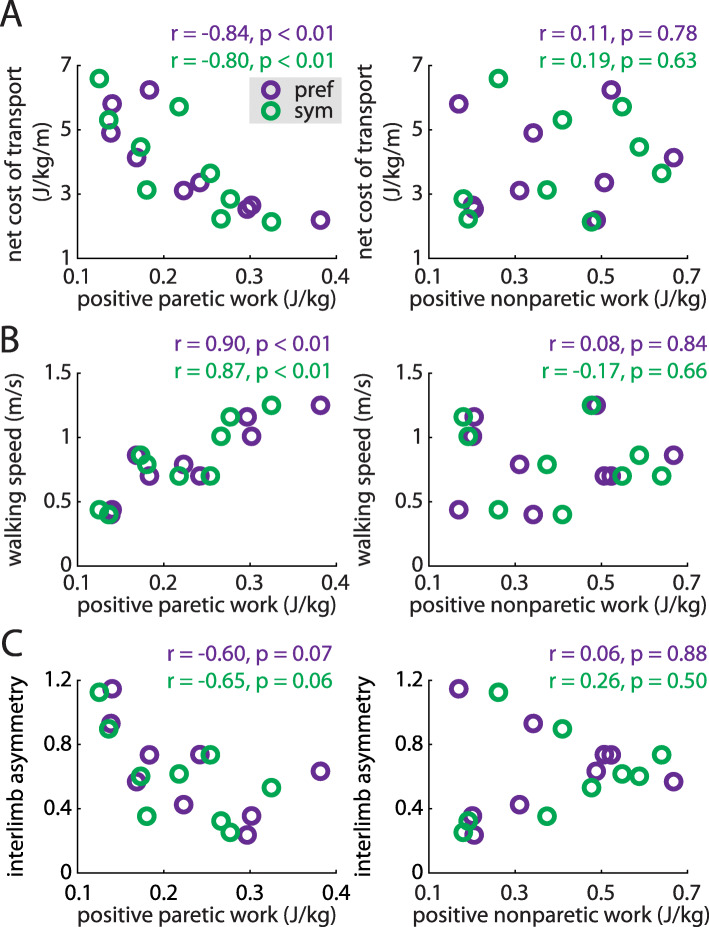
Positive paretic work correlates strongly with the net metabolic cost of transport (**a**, left), walking speed (**b**, left) during both preferred walking (purple) and symmetric stepping (green). The correlation between positive paretic work and IA shows a similar trend but is not statistically significant in either condition (**c**, left). Positive nonparetic work is not significantly associated with any of these variables (**a**, **b** and **c**, right) during either condition

We then assessed whether the positive and negative work done by each leg across the gait cycle were correlated with cost of transport, gait speed, or IA during preferred walking and symmetric stepping. Positive paretic work was significantly correlated with decreased cost of transport during both conditions (preferred walking: *r* = − 0.84, *p* < 0.01; symmetric stepping: *r* = − 0.80, *p* < 0.01; Fig. 6a, left); positive nonparetic work was not (both *p* > 0.63, Fig. 6a, right). Positive paretic work was also significantly correlated with increased walking speed (preferred walking: *r* = 0.90, *p* < 0.01; symmetric stepping: *r* = 0.87, *p* < 0.01; Fig. 6b, left) whereas positive nonparetic work was not (both *p* > 0.66, Fig. 6b, right). Finally, positive paretic work was negatively correlated with decreased IA during preferred walking and symmetric stepping, though these trends did not reach statistical significance (preferred walking: *r* = − 0.62, *p* = 0.07; symmetric stepping: *r* = − 0.65, *p* = 0.06; Fig. 6c, left). Positive nonparetic work was not significantly correlated with IA during either condition (both *p* > 0.50; Fig. 6c, right). We did not observe significant correlations between negative paretic or nonparetic work and cost of transport, walking speed, or IA during either condition (all *p* > 0.10).

## Discussion

Gait kinematics, kinetics, and cost of transport changed very little when persons post-stroke used visual feedback to improve step length symmetry. Although participants were not provided specific instructions on how to change their step lengths to attain symmetry, participants significantly lengthened the shorter step and shortened the longer step to improve step length symmetry. Even when walking with more symmetric steps, participants exhibited considerable kinematic asymmetry, impaired paretic propulsion during late paretic stance, and excessive compensatory vertical movement of the COM during late paretic stance and nonparetic single support [4, 31, 32]. Deficits in positive paretic work were also unaffected by improvement in step length symmetry and were correlated with cost of transport and walking speed. These findings reveal that step length symmetry improvement does not necessarily result in positive changes elsewhere in the gait pattern after stroke. It is critical that interventions do not merely aim to restore step length symmetry but rather address the underlying impairments in gait mechanics and/or control.

We do not necessarily intend for these findings to be interpreted as an indictment on step length asymmetry as a target of post-stroke gait rehabilitation. Interventions that improve step length asymmetry have shown coincident improvement in gait speed [15] and decreased cost of transport [19]. However, step length asymmetry arises from a complicated series of deficits [4, 5, 22]. We propose that it is not so important *that* step length symmetry is restored, but rather *how* step length symmetry is restored – i.e., how underlying deficits in kinematics, kinetics, or muscle activation that cause step length asymmetry are addressed – that will facilitate gait improvement more broadly. For example, interventions that restore step length symmetry by improving paretic propulsion [19] have shown potential for driving meaningful improvement in post-stroke gait. Furthermore, in this study, persons post-stroke tended to improve step length symmetry by simultaneously shortening the longer step and lengthening the shorter step. In clinical practice, shortening the longer step is unlikely to be a relevant goal. Lengthening the shorter step may have different biomechanical and metabolic consequences than shortening the longer step [21], and it will be important for future studies to examine these effects.

How might we design interventions to restore step length symmetry and decrease cost of transport? The between-participant correlational analyses showing positive paretic work to be correlated with cost of transport and gait speed (and, to a lesser extent, IA) do not establish causal relationships between these variables. However, they do highlight positive paretic work as an important topic of future investigation. Although mechanical work is not related to the cost of transport in all walking conditions [33], a substantial portion of the cost of transport can be attributed to the mechanical work generated by the legs on the COM during step-to-step transitions [26, 34–36]. Persons post-stroke generate more positive work with the nonparetic leg than the paretic leg [4, 27, 28, 31, 37], and this deficit is influenced by the lack of sufficient paretic propulsion during the step-to-step transition occurring during late paretic stance [32, 38]. Importantly, this propulsion must also occur at the appropriate timing [39]. Interventions that improve paretic propulsion and extend paretic stance may then enhance the ability of the paretic leg to generate positive work and facilitate a faster, more symmetric, less effortful walking pattern.

How might we reconcile the inability to generate positive paretic work with an increase in cost of transport after stroke? Prior studies have explained how the reduced positive work generated via paretic propulsion reverberates throughout the walking pattern. Paretic stance time is shortened relative to nonparetic stance [4], and impaired paretic propulsion decreases the energy transmitted to the leg to initiate swing [31, 40–42]. This necessitates compensatory mechanics to facilitate paretic leg swing. Often, vaulting (vertical COM movement generated primarily by the nonparetic leg) occurs to lift the paretic foot from the floor. This elevates vertical COM velocity and increases the positive work done to raise the COM (rather than direct it forward) during late paretic stance [8, 27, 28, 31]. Persons post-stroke then often use a sequence of pelvic rotation, hip hiking, and hip circumduction to swing the leg and clear the foot [4, 31]. This slow paretic leg swing prolongs nonparetic stance, increasing the positive nonparetic work done during single support [8].

The inability to generate sufficient paretic propulsion during late stance thus requires 1) increased positive nonparetic work during single support and late paretic stance, often in the vertical direction, or 2) compensatory demands on the nonparetic leg that can be lessened by decreasing gait speed [43]. Either of these factors could result in an elevated cost of transport, and the altered mechanics persisted across both preferred walking and symmetric stepping. Fortunately, many interventions – including fast walking [44–46], functional electrical stimulation of the plantarflexors [46], and split-belt treadmill walking [20, 47] – show promise for improving paretic propulsion [38]. These interventions target paretic propulsion through combinations of improving ankle power generation and increasing paretic limb extension during late stance (i.e., trailing limb angle [41, 48]). Improving propulsion by increasing ankle power is also a common goal in robotic designs [39, 49–51], and multiple interventions that target paretic propulsion have resulted in improved step length symmetry [11, 19].

Our study was not without limitations. We focused on the relationship between step length symmetry and cost of transport during only a single testing session and only by using visual feedback to improve step length symmetry. Improved step length symmetry without changes in the underlying gait mechanics may affect cost of transport differently if the improved symmetry is achieved via long-term training. The participants included here exhibited mild-to-moderate gait dysfunction and were in the chronic phase post-stroke. These results may not extrapolate to more impaired patients or patients in the acute phase post-stroke. While participants were instructed to avoid holding the handrails as much as possible during the task, not all participants were able to do so. We did not use instrumented handrails and could not quantify the impact of holding on to the handrails on IA or mechanical work done.

## Summary

Persons post-stroke improve step length symmetry using energetically expensive, asymmetric walking patterns that are largely similar to their preferred gait. The similarity in cost of transport observed during preferred walking and symmetric stepping can be explained by the persistence of aberrant gait mechanics (specifically, impaired ability to generate positive paretic work) regardless of step length symmetry. Our findings suggest that future interventions should target the gait deficits that underlie step length asymmetry rather than step length asymmetry in isolation.

## Data Availability

The datasets used and/or analyzed during the current study are available from the corresponding author on reasonable request.
